# Effect of Chestnut Tannins and Short Chain Fatty Acids as Anti-Microbials and as Feeding Supplements in Broilers Rearing and Meat Quality

**DOI:** 10.3390/ani9090659

**Published:** 2019-09-05

**Authors:** Federica Mannelli, Sara Minieri, Giovanni Tosi, Giulia Secci, Matteo Daghio, Paola Massi, Laura Fiorentini, Ilaria Galigani, Silvano Lancini, Stefano Rapaccini, Mauro Antongiovanni, Simone Mancini, Arianna Buccioni

**Affiliations:** 1 Dipartimento di Scienze e Tecnologie Agrarie, Alimentari, Ambientali e Forestali, University of Florence, Piazzale delle Cascine 18, 20144 Firenze, Italy (G.S.) (M.D.) (I.G.) (S.L.) (S.R.) (A.B.); 2 Dipartimento di Scienze Veterinarie, University of Pisa, Viale delle Piagge 2, 56124 Pisa, Italy (S.M.) (S.M.); 3 Istituto zooprofilattico Sperimentale della Lombardia e dell’Emilia Romagna-Sezione di Forlì, via don Eugenio Servadei 3/E-3/F, 47122 Forlì, Italy (G.T.) (P.M.) (L.F.); 4 Gruppo Mauro Saviola srl, Viale Lombardia 29, 46019 Viadana MN, Italy; 5 Centro Interdipartimentale di Ricerca per la Valorizzazione degli Alimenti, Largo Brambilla, 3, 50134 Firenze, Italy

**Keywords:** antibiotic, hydrolysable polyphenol, monoglyceride, pathogen, poultry feeding

## Abstract

**Simple Summary:**

The poultry industry needs to replace antibiotics with natural or synthetic compounds able to overcome problems linked to the development of bacterial resistance. Tannins and short chain fatty acids are valid alternatives to contrast the growth of pathogens. However, tannins may induce detrimental effects on animal performances, especially in monogastrics, causing damage on gut villi. In contrast, short chain fatty acids are very efficient in influencing positively the morphology of small intestine wall. Hence, the aim of this trial was to develop a feeding strategy for broiler rearing, based on the use of chestnut tannins and short chain fatty acids administered as blends. No differences in animal performances or in meat quality were found among feeding groups. The results suggested that the mix of these supplements did not have negative effects on the productive performances, representing a promising alternative to antibiotics. However, further investigation is needed to better understand the effects of these supplements on animals in stress conditions.

**Abstract:**

Chestnut tannins (CT) and saturated short medium chain fatty acids (SMCFA) are valid alternatives to contrast the growth of pathogens in poultry rearing, representing a valid alternative to antibiotics. However, the effect of their blends has never been tested. Two blends of CT extract and Sn1-monoglycerides of SMCFA (SN1) were tested in vitro against the proliferation of *Clostridium perfringens*, *Salmonella typhymurium*, *Escherichia coli*, *Campylobacter jejuni*. The tested concentrations were: 3.0 g/kg of CT; 3.0 g/kg of SN1; 2.0 g/kg of CT and 1.0 g/kg of SN1; 1.0 g/kg of CT and 2.0 g/kg of SN1. Furthermore, their effect on broiler performances and meat quality was evaluated in vivo: one-hundred Ross 308 male birds were fed a basal diet with no supplement (control group) or supplemented with CT or SN1 or their blends at the same concentration used in the in vitro trial. The in vitro assay confirmed the effectiveness of the CT and SN1 mixtures in reducing the growth of the tested bacteria while the in vivo trial showed that broiler performances, animal welfare and meat quality were not negatively affected by the blends, which could be a promising alternative in replacing antibiotics in poultry production.

## 1. Introduction

Conventional antimicrobial agents are commonly used in the poultry industry to control diseases and to prevent the mortality of birds. However, this approach conflicts with the worldwide aim to eliminate antibiotics in animal feeding. Indeed, the use of pharmaceuticals as preventing tools against pathogens has contributed to the acquisition of bacterial resistance. Moreover, problems for dejection disposal occur, due to the presence of residual antibiotics. Therefore, the poultry industry needs alternatives able to replace drugs with natural compounds or with synthetic compounds able to simulate natural molecules [1].

Polyphenols from plant kingdom are efficient antimicrobials, even if major differences can be noted. Indeed, their efficacy is affected by the solubility, which is strongly linked to the molecular structure. Among the others, chestnut tannins (CT) are hydrolysable and water-soluble compounds. Their antimicrobial activity has been previously demonstrated in poultry by Tosi et al. [2], while Redondo et al. [3] reported that *Clostridium perfringens* is unable to develop resistance against hydrolysable tannins, compared to antibiotics as avilamycin or bacitracin. However, the use of tannins in animal feeding, with particular reference to monogastrics, is discouraged for their potential anti-nutritional effects [4]. The reason is their ability in binding proteins, lowering feed intake and digestibility [4,5,6]. Hence, to evaluate the inclusion level of polyphenols is extremely important to avoid detrimental effects on animal welfare and performances [7,8].

Furthermore, literature shows that free saturated short-medium chain fatty acids (SMCFA; from C4:0 to C12:0) can protect the gut against several pathogenic bacteria [9,10,11], but their employment is limited because they are quickly absorbed in the jejunum [12,13,14]. Similarly, Sn2-monoglycerides are easily carried from the gut into the blood stream [6,15]. A hypothetical alternative could be represented by synthetic monoglycerides, because the industrial synthesis occurs under kinetic control of the reaction and the end-products are Sn1-substituted monoglycerides. These molecules, according to a not-natural structure, are little absorbed at the gut level, where they can exert antimicrobial effects against pathogens [16].

Several studies have been published on the efficiency of CT and synthetic monoglycerides in exercising antimicrobial activities and in ameliorating animal performances when used separately [12,13,14,17]. Nevertheless, no information is available in literature on the effect of blends of CT and Sn1-monoglycerides (SN1) of SMCFA as antimicrobials against the proliferation of *C. perfringens*, *Salmonella typhymurium*, *Escherichia coli*, *Campylobacter jejuni* and as dietary supplement in poultry diets. Hence, the aims of this trial were: (i) to test a possible synergic antimicrobial activity of two blends obtained from a mixture of two commercial supplements (i.e., CT extract from *Castanea sativa* Mill. and SN1) by an in vitro study, then (ii) to evaluate the effect of the same mixtures on broiler performance and meat quality.

## 2. Materials and Methods

### 2.1. Chestnut Hydrolysable Tannins and Sn1-Monoglycerides Composition

Chestnut tannins were extracted from the wood of *C. sativa* Mill. by distillation with water flow (Saviotanfeed^®®^, Gruppo Mauro Saviola srl Radicofani, Siena, Italy) and contained 750 g/kg (on dry matter basis, DM) of equivalent tannic acid. The chromatographic characterization of this lot of CT extract is reported in Bargiacchi et al. [18]. Sn1-monoglicerydes contained a mix of SMCFA from C4:0 to C12:0 (Silohealth^®®^, Silo SpA, Firenze, Italy). The glycerides and fatty acid (FA) profile of SN1 was determined according to Christie [19] and it is shown in Table 1. These supplements were the same used in the microbiological assay and during the in vivo trial.

### 2.2. Microbiological Assay

The microbiological assay was carried out according to Elizondo et al. [20] modified as described below. The microorganisms used in this study were the following: *C. perfringens* NetB positive (strain number 191999/2014), isolated from broiler chickens affected by necrotic enteritis; *S. typhimurium* (strain number 198306/2014), isolated from viscera of egg-table layers; *E. coli* serotype O45 (strain number 184049/2014), isolated from broiler chickens affected by avian colibacillosis; *C. jejuni* (strain number 18818/2015), isolated from the skin of broiler chickens. The bacterial strains were isolated and identified using the standard procedures adopted by Istituto Zooprofilattico Sperimentale della Lombardia e dell’Emilia Romagna (section located in Forlì, Italy) and maintained on slants with heart infusion agar (HIA; Becton Dickinson GmbH, Germany) at +4 °C. To ensure culture purity, before the assays, a sample of *C. perfringens* culture was streaked on blood agar base (Oxoid Ltd., Basingstoke, UK) with 5 g/100 g of sheep blood and incubated overnight at 37 °C under anaerobic conditions (GENbag anaer, bioMérieux S.A., Marcy l’Etoile, France). As similar, the samples of *E. coli* and *S. typhimurium* cultures were streaked on Hektoen Enteric Agar (Becton Dickinson GmbH, Germany) and incubated overnight at 37 °C. The samples of *C. jejuni* cultures were streaked on modified charcoal cefoperazone deoxycholate (mCCD) agar (Oxoid Ltd., Basingstoke, UK) and incubated at 44 °C for 48 h in a microaerobic atmosphere (GENbag microaer, bioMérieux S.A., Marcy l’Etoile, France). Then, one colony of each strain was grown in Brain Heart Infusion (BHI) broth (Becton Dickinson GmbH, Germany) and incubated overnight at 37 °C (under anaerobic conditions for *C. perfringens*; microaerobic atmosphere at 44 °C for *C. jejuni*) and then titrated. For this purpose, 10-fold serial dilutions of each suspension were carried out in Buffered Peptone Water (Oxoid Ltd., Basingstoke, UK); each dilution was streaked on specific media and incubated overnight at 37 °C (under anaerobic conditions for *C. perfringens*; microaerobic atmosphere at 44 ℃ for *C. jejuni*). Based on the titration results, each bacterial suspension was diluted in BHI broth to obtain a concentration of 2 × 10^3^ CFU/mL and then used as inoculum in the antibacterial test described below.

The antimicrobial activity in vitro assay was carried out according to Basri and Khairon [21] modified as follow. Five milliliters of each bacterial suspension (described above) was mixed with 5.0 mL of the following concentrations (in BHI broth, expressed as *w*/*v*) of the tested compounds: 6.0 g/kg of CT (T group), 6.0 g/kg of SN1 (S group), 4.0 g/kg of CT + 2.0 g/kg of SN1 (TS group) and 4.0 g/kg of SN1 + 2 g/kg of CT (ST group). Untreated tubes containing 5.0 mL of each bacterial suspension and 5.0 mL of BHI broth served as control (C group). This procedure resulted in a final concentration of the bacterial inoculum of 1 × 10^3^ CFU/mL and in the following final concentrations (*w*/*v*) of the tested compounds: 3.0 g/kg of CT (T group), 3.0 g/kg of SN1 (S group), 2.0 g/kg of CT + 1.0 g/kg of SN1 (TS group) and 2.0 g/kg of SN1 + 1.0 g/kg of CT (ST group). The concentrations of supplements in T and in S groups were chosen according to the producers’ guidelines. The ratio among CT and SN1 in the two blends was decided referring to preliminary studies [2,22]. The mixtures were incubated at 37 °C (under anaerobic conditions for *C. perfringens*; microaerobic atmosphere at 44 °C for *C. jejuni*). Each suspension was assayed at 0.5 h, 3 h and 24 h of incubation by making 10-fold dilutions in Buffered Peptone Water (Oxoid Ltd., Basingstoke, UK), streaking 100 µL of each dilution on specific media and incubated as described above (adapted from Elizondo et al. [20]). The viable bacterial counts (expressed as CFU/mL) of the tested compounds for each concentration were compared with the bacterial counts obtained in the C group. The assays were repeated three times and results were expressed as the average values.

The microbial growth rate was calculated as ratio: Δ_Conc_ (CFU/mL)/Δ_Time_ (h), where Δ_Conc_ is calculated as difference among microbial concentrations at the ranges of 0.5–3 h or 3–24 h and Δ_Time_ is the related interval between the sampling times (0.5–3 h; 3–24 h).

### 2.3. In Vivo Trial

#### 2.3.1. Animals

Animal handling was in accordance with Italian Government guideline (D.lgs 26/2014, protocol number 232/2016PR). One hundred one-day-old Ross 308 male chicks were provided by a local hatchery (Incubatoio Settecrociari, Forlì-Cesena, Italy), where they were vaccinated against Marek’s disease, infectious bronchitis and Newcastle disease. Birds were allotted in 20 pens (5 animals per pen singularly identified by ring) and randomly assigned to one of the 5 experimental diets (4 pens each diet). The feeding groups, summarized in Table 2, were: control group (C group), fed with a basal diet containing tannins free and SN1-monoglycerides of SMCFA free ingredients (Table 3); T group, fed with the basal diet supplemented with 3.0 g/kg on DM of CT; S group, fed with the basal diet supplemented with 3.0 g/kg on DM of SN1; TS group, fed with the basal diet supplemented with 2.0 g/kg on DM of CT and 1 g/kg on DM of SN1; ST group, fed with the basal diet supplemented with 1.0 g/kg on DM of CT and 2.0 g/kg on DM of SN1. The diets were formulated according to animal requirements (NRC, 1994) with 3 periods of growth: starter (0–12 days), grower (13–21 days) and finisher (22–35 days). The dosage of CT, SN1 and of their blends was the same used in the microbiological assay. Animals were fed *ad libitum* and had free access to water for all the 35 days of the trial. Every week, the animals from each pen were individually weighted. The individual feed intake was registered weekly for each pen and calculated dividing the total amount consumed by the number of animals present in the pen (the approved protocol did not allow the use of individual pens). Feed efficiency was calculated as estimated ratio of the individual feed intake/registered individual weight gain for each group.

#### 2.3.2. Diet Proximate Analysis

Diets were analyzed for proximate profile as follows: crude protein (CP), ether extract (EE), crude fiber (CF) and ash were determined according to the AOAC methods 976.06, 920.39, 962.09 and 942.05, respectively (AOAC 1995). Neutral detergent fiber (NDF) was determined according to van Soest et al. [23], using heat stable amylase and sodium sulphite, and expressed inclusive of residual ash. Metabolizable Energy (ME) was estimated from feed tables according to Sauvant et al. [24]. The chemical and nutritional profile of the basal diets are reported in Table 4.

#### 2.3.3. Physical and Chemical Analysis

All carcasses were evaluated for dressing out and major traits. Breast meat from three animals of each pen was sampled for color analysis, antioxidant capacity, and oxidative status as follows.

- Color analysis. The samples of breast were poured into a clean glass petri dish to be evaluated for color using the portable spectrophotometer (Minolta CR 200 Chroma Meter4, Konica Minolta Chiyoda, Tokyo, Japan, calibrated using a standard yellow calibration tile, model CRA471). The top of the Chroma Meter measuring head was placed flat against the surface of the meat and the reflective color was determined from the average of three consecutive pulses from the optical chamber of the spectrophotometer. Data are reported in the L* a* b* color notation system [25] with L* axis representing lightness, the a* axis representing the red-green color axis (redness) and the b* axis representing the blue-yellow (yellowness) color axis. The numerical total color difference (ΔE2000) among samples was calculated by the formula proposed by Mokrzycki and Tatol [26].
- Antioxidant capacity. Meat samples (5.0 g) were extracted with ethanol as reported by Mancini et al. [27]. The filtrate was used to measure ABTS (2,2-azinobis-(3-ethylbenzothiazoline-6-sulfonic acid)) reducing activity, DPPH (1,1-diphenyl-2-pircydrazyl) radical scavenging activity and ferric reducing ability (FRAP), as reported by Mancini et al. [27] and modified from Blois [28], Re et al. [29] and Descalzo et al. [30]. Results were expressed as mmol of Trolox equivalent per kg of fresh meat for ABTS and DPPH methods and as mmol of Fe^++^ equivalent per kg of fresh meat for FRAP determination.
- Oxidative status of meat. Meat samples (5.0 g) were considered for TBARS (thiobarbituric acid-reactive substances) determination. TBARS were measured to determinate malondialdehyde (MDA) levels, according to the method described by Ke et al. [31] and modified by Dal Bosco et al. [32]. Briefly, the meat samples were homogenized with a water solution of trichloroacetic acid (7.5% *w*/*v*) and diethylenetriaminepentaacetic acid (0.1% *w*/*v*). After centrifugation and filtration, the solutions were reacted with a water solution of 2-thiobarbituric acid (0.288% *w*/*v*) and heated in a water bath at 95 °C for 45 min. The absorbance of the samples was determined at 532 nm (V-530 Jasco International, Milan, Italy) and a calibration curve was plotted with TEP (1,1,3,3-tetraethoxypropane; 0–15 μM, final concentrations) to obtain the MDA concentration. Results were expressed as mg of MDA-equivalents per kg of fresh meat.

#### 2.3.4. Statistical Analysis

Data related to bacterial counts were expressed as log10 (CFU/mL) and normalized according to Snedecor and Cochran [33]. Data related to microbial growth rate were processed as completely randomized design with repeated measures using the MIXED procedure of SAS [34]:

Y_ijkl_ = μ + T_i_ + D_j_ + I_k_(D) + (T × D)_ij_ + e_ijkl_, (1)

where y_ijkl_ is the observation; μ is the overall mean; D_j_ is the fixed effect of treatment (j = 1 to 5); T_i_ is the fixed effect of assaying time (i = 1 to 3); I_k_ is the random effect of the replicate nested within the treatment (k = 1 to 3); (T × D)_ij_ is the interaction between treatment and assaying time and e_ijkl_ is the residual error. The covariance structure was compound symmetry, which was selected based on Akaike’s information criterion of the mixed model of SAS [34]. The statistical significance of the treatment effect was tested against variance of bacterial cultures nested within treatment, according to repeated measures design theory [35]. Multiple comparisons among means were performed using the Tukey’s test.

One-hundred animals divided in 5 groups is the minimum number of animals in order to obtain significant differences among treatments according to the power analysis based on alpha 0.05 beta 0. 08 [33]. Data related to the feed intake, weight gain, feed efficiency of each period, were processed as completely randomized design with repeated measures using the MIXED procedure of SAS [34]:

Y_ijkl_ = μ + T_i_ + D_j_ + I_k_(D) + (T × D)_ij_ + e_ijkl_, (2)

where y_ijkl_ is the observation; μ is the overall mean; D_j_ is the fixed effect of treatment (i = 1 to 5); T_i_ is the fixed effect of assaying time (j = 1 to 5); I_k_ is the random effect of the replicate nested within the treatment (k = 1 to 5); (T × D)_ij_ is the interaction between treatment and assaying time and e_ijkl_ is the residual error. The covariance structure and the statistical significance were tested as described above.

The data related to feed intake, weight gain, feed efficiency of the whole period, physical and chemical parameters of meat, dressing out and the major carcass traits of slaughtered birds were analysed by one-way ANOVA, keeping the factor “diet” as the fixed one [34]:

y_ij_ = μ + D_i_ + e_ij_, (3)

where y_ij_ is the observation; μ is the overall mean; D_i_ is the diet (i = 1 to 5) and e_ij_ is the residual error. Multiple comparisons among means were performed using the Tukey’s test. Probability of significant effect due to experimental factors was fixed for *p* < 0.05.

## 3. Results and Discussion

### 3.1. Microbiological Assay

**Figure 1:**
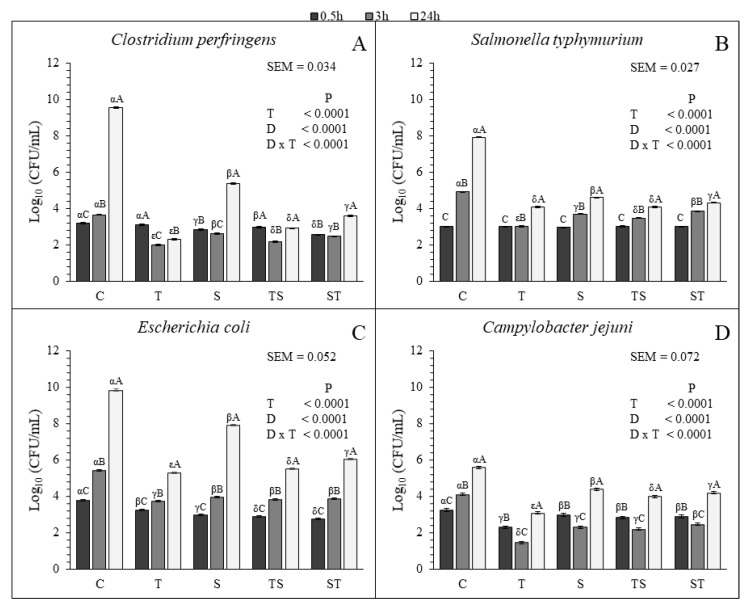
Results of microbial in vitro assay for *Clostridium perfringens* (**A**), *Salmonella typhymurium* (**B**), *Escherichia coli* (**C**), *Campylobacter jejuni* (**D**) (data are reported as Log10 (CFU/mL)). C, control; T, 3.0 g/kg of chestnut tannin; S, 3.0 g/kg of Sn1-monoglycerides; TS, 2.0 g/kg of chestnut tannin added to 1.0 g/kg of Sn1-monoglycerides; ST, 1.0 g/kg of chestnut tannin added to 2.0 g/kg of Sn1-monoglycerides. Concentration are expressed as *w*/*v*. SEM, Standard Error Mean. The probability of significant effect due to experimental factors is reported as: α, β, γ, δ, ε for the treatments (means with different Greek superscripts are significantly different (*p* < 0.05)); A, B, C for the sampling time (means with different Latin superscripts are significantly different (*p* < 0.05)).

All the treatments were efficient in decreasing the bacterial growth of each species compared to the control (Figure 1). The T resulted the most effective treatment in controlling the growth of each bacterial species at 3 h and 24 h. For the other treatments, the behavior of the tested bacteria was different. *C. perfringens* and *S. typhymurium* resulted more sensitive to the TS than to the ST and S at 3 h and 24 h. At 3 h no significant differences were found for the S, TS and ST for *E. coli* but at 24 h the TS was more efficient in limiting the growth compared to the other two treatments (Figure 1C). No significant different growth was observed for *C. jejuni* with S and TS at 3 h. However, at the same sampling time, the growth with ST was higher than the growth with S and TS. At 24 h the growth of *C. jejuni* was lower with the TS than with the S and ST (Figure 1D).

All the treatments lowered the growth rate of each microbial species, except for *C. jejuni* that did not show significant decreasing with the tested compounds between 3 h and 24 h, compared to the control. Furthermore, both *C. perfringens* and *C. jejuni* decreased in all the treatments between 0.5 h and 3 h, and increased between 3 h and 24 h (Table 5 and Figure 1). This observation suggested that the bactericidal effect of CT and SN1, supplied alone or in combination, is stronger at the beginning of the treatment. For *E. coli* and *S. typhymurium* only a bacteriostatic effect was observed in the treated cultures (Table 5 and Figure 1).

Our results are in accordance with Tosi et al. [2] and Redondo et al. [3] who demonstrated that the CT can inhibit the growth of *C. perfringens*. The antimicrobial activity of tannins seems to be due to their ability to bind microbial enzymes and proteins, in ion deprivation and in inhibiting the topoisomerase, fundamental for the DNA replication [36,37,38]. Moreover, Ramìrez et al. [6] and Timbermont et al. [10] showed that the SN1 of SMCFA were efficient in controlling the growth of *S. typhimurium* and *C. perfringens*, respectively, consistent with our study. Their antimicrobial effect was explained with their ability to penetrate through the bacterial wall, because of their affinity with lipoteichoic acid, present in microbial membrane. Their ability to destroy the inner the membrane is probably due to their compatibility with hydrophilic and hydrophobic moieties [6,39,40,41]. These results suggest that the CT and SN1 alone or in combination could be useful to control the proliferation of pathogenic bacteria tested in this trial. Hence, these molecules could represent a valid alternative to antibiotics both used alone or in mixture.

### 3.2. In Vivo Trial

No differences among the groups were found for feed intake (Table 6), both in each single growth period and in the whole period of bird life, showing that the supplementation with CT and SN1 blends did not affect the palatability of the diets. No differences were found for weight gain and feed efficiency among groups, suggesting that the blends of CT and SN1, at the inclusion level adopted in this study, did not interfere with nutrient absorption, with respect to the single supplementation or to the control diet (Table 6). Hence, no synergic effect was found when the CT and SN1 mixtures were included in the diets. The results showed a higher feed/gain ratio during the first two weeks, compared to the growing and finisher periods. Usually, young chicks have higher feed efficiency than old birds. This trend could be due to an adaptation period of birds to the rearing condition because also the control group, fed with only the basal diet, did not show significant differences with the other feeding groups. The literature reports information on the effect of CT and SN1 when they are included alone in the diets, but few data are available on the effect of blends composed by a mixture of tannin extracts and monoglycerides. The results of this trial are in accordance with Jamroz et al. [42] and Antongiovanni et al. [13,14,43] who studied respectively the effect of CT (inclusion level of 0, 250, 500 and 1000 mg/kg on DM) and of several monoglycerides (inclusion level of 200, 350, 500 mg/kg of DM) separately, as dietary supplementation on the performance and histological characteristics of the intestine wall in chickens. No impairment of the growth performance emerged, despite a slight modification on the small intestine wall, due to the introduction in the diet of chestnut tannins and monobutyrin (CT degrades enterocytes while monobutyrin modifies positively villi, microvilli and crypts), was observed. Moreover, previous results, reported by Schiavone et al. [17], showed that the inclusion of a natural extract of chestnut wood did not affect the apparent digestibility of CP and that this supplement had a positive effect on average daily gain and feed intake in the first two weeks of addition. For monoglycerides, especially with butyric acid, the literature confirms that they can ameliorate growth performances and health in broilers [12,13,14]. In contrast, for the CT, several studies reported that polyphenols reduce protein digestibility in monogastrics, decreasing the productive performances in accordance with a lower availability of this nutrient for the animal nutritional requirements [5,44,45,46]. In particular, tannins stimulate hypersecretion of endogenous enzymes leading to losses of sulphur aminoacids in poultry species [47,48,49]. The inconsistence of the results reported in many papers, including those shown here, is probably due to the kind of tannin used as dietary supplement, the animal species and the dietary dose formulation. In this study, by an accurate observation, an astringent effect of tannin has been noted in T, TS and ST groups, whose litters resulted drier than the litters of S group (at 35th day: C = 1; T = 0; TS = 0; ST = 0 and S = 1).

No significant differences in carcass quality were found among groups (Table 7), except for the liver that was smaller in the animals fed the T and S diets than the other feeding groups. This result is consistent with the findings reported by Jamroz et al. [42] and Antongiovanni et al. [13] who noted that the supplementation with polyphenols or the monoglycerides of butyric acid did not affect carcass quality, even though monoglycerides represented an energy source for animal growth and tannins are considered to be antinutritive. Unfortunately, in literature, no information is available on blends of CT and SN1, which were not able to affect carcass traits at the tested levels in our study.

Changes in the a* and b* values are related to changes in meat color because these parameters are markers of browning [50]. Where the SN1 was present in the diet alone an increase of the L* and b* values occurred (Table 8). Specifically, the S group showed the highest L* and b* values and it significantly differed compared to the other groups. Despite the statistical differences, ΔE calculation for the samples of chicken breast can be useful to understand how the values of color can be perceived by human eyes (Table 9). As suggested by Mokrzycki and Tatol [26], a standard observer is able to see the difference in color as follows: 0 < ∆E < 1 observer does not notice the difference, 1 < ∆E < 2 only experienced observers can notice the difference, 2 < ∆E < 3.5 unexperienced observer also notices the difference, 3.5 < ∆E < 5 clear difference in color is noticed, ∆E > 5 observer notices two different colors. Regarding the ∆E, from Table 9 it emerged that the T and TS were the most similar groups (1 < ∆E < 2), while the ST assumed a 2 < ∆E < 3.5 when compared with C, T, and TS. Interestingly, the S group was found to be the only one with a ∆E > 5 when compared to the other experimental groups, thus underlining that Sn1 monoglycerides strongly impacted meat coloration. Whether this modification could be accepted or not by consumers should be further investigated.

Data reported in this study showed that both the tested blends of CT and SN1 could be utilized as dietary supplements without impairing animal welfare, growing performances and meat quality, thus representing valid alternatives to antibiotics in poultry rearing. Contrariwise, the S diet deeply modified the color of breast meat which could result in a modification of consumers’ acceptance.

In this trial, data related to the antioxidant status of the breast meat did not show significant differences among the groups (Table 10). Several studies demonstrated the antioxidant power of tannins and of polyunsaturated fatty acids but not for SMCFA, because of the lack of double bounds on carbon chain [51,52,53]. Indeed, several authors reported that ellagic tannins in humans and rats are gradually metabolized by the intestinal microbiota to produce different metabolites with antioxidant effects [54,55,56,57]. Luciano et al. [53] found that the inclusion at 8.96% (DM basis) of quebracho tannins in lamb diet produced an improvement in the antioxidant status of *Longissimus dorsi* muscle, measured as both its ferric reducing ability and its radical scavenging ability. Other authors reported similar results in beef meat [30,58,59]. In contrast, Gladine et al. [60] found no effect of polyphenols in rat muscle for the radical scavenging activity. The inability of many polyphenols to be metabolized by the gastrointestinal tract of animals is strongly linked to their molecular structure and solubility, the dose of inclusion and the animal species.

Nowadays, in conventional and intensive poultry production, antibiotics are used to control diseases and to prevent the mortality of birds, responsible for a huge economical loss. This approach conflicts with the sustainability of animal productions because several issues about the development of bacterial resistance, dejection disposal, food safety and human health occur. Therefore, the poultry industry needs alternatives able to replace antibiotics with natural or synthetic compounds able to simulate natural molecules. Chestnut tannins are a by-product of wood industry because they are obtained by distillation of wood used in the building industry. SN1 is obtained by recycling glycerol derived from biodiesel production. Hence, CT and SN1 are part of the concept of bio-economy. Moreover, FAO reported that livestock support the livelihoods and food supply of almost 1.3 billion people, being one of the fastest growing areas of the agricultural economy in the world. In developing countries, poultry production plays an important role in food, and pathogen proliferation represents an important public health problem that cannot be underestimated. At the same time, environmental sustainability must be ensured [61]. Data reported in this study showed that CT, SN1 or their blends could represent a valid alternative to antibiotics in poultry rearing. Although literature shows several studies in which tannins exert antinutritional effects in monogastric, in the present in vivo trial, no detrimental effects were observed on animal welfare, performance or meat quality. However, it is well known that the kind of polyphenols and the dietary inclusion level are fundamental to explain the biological and nutritional effects of dietary tannins. Besides, the literature shows unequivocal positive effects of SN1 monoglycerides in protecting gut from pathogens, by providing energy to enterocytes and by favoring the development of gut villi [12,13,16]. Hence, the blends of CT and SN1 could represent a good compromise among antimicrobial activities, animal gut protection, meat quality and production sustainability.

## 4. Conclusions

The in vitro study suggested that blends of CT and SN1 could be efficient against the proliferation of *C. perfringens*, *S. typhymurium*, *E. coli* and *C. jejuni*. Additionally, the in vivo trial suggested that the mixture of these supplements did not have negative effects on animal productive performances, representing a promising alternative to antibiotics. Considering all the recorded information about in vitro antimicrobial effectiveness and the broilers growing performances, meat color and the overall antioxidant capacity of meat, both the TS and ST tested blends might be good alternatives to antibiotics in the poultry sector. However, further investigation is needed to better understand the effects of these supplements on animals in stress conditions.

## Figures and Tables

**Table 1:** Lipid profile of Sn1-monoglycerides.

**Glycerides Composition g/100 g of Product**	
monoglycerides	95.0
diglycerides	4.9
triglycerides	0.1
**Fatty Acid Composition g/100 g of Total Lipids**	
C4:0	50.0
C6:0	12.5
C8:0	12.5
C10:0	12.5
C12:0	12.5

**Table 2:** Experimental design.

Group	Number of Animals Per Pen	Number of Pens	Diet
C	5	4	basal diet
T	5	4	basal det + 3 g/kg DM of chestnut tannin extract
S	5	4	basal det + 3 g/kg DM of SN1 monoglicerides
TS	5	4	basal det + 2 g/kg DM of chestnut tannin + 1 g/kg DM of SN1 monoglicerides
ST	5	4	basal det + 1 g/kg DM of chestnut tannin + 2 g/kg DM of SN1 monoglicerides

**Table 3:** Ingredient composition (g/kg of DM) of basal diets formulated according to growing periods.

Ingredients	Starter (0–12 d)	Grower (13–21 d)	Finisher (22–42 d)
Maize	330.0	360.0	380.0
Wheat	240.0	240.0	230.0
Soy bean meal	220.0	220.0	200.0
Animal fat	39.0	43.0	60.0
Maize gluten feed	30.0	22.0	15.0
Hydrolysed protein	33.0	10.0	10.0
Sunflower meal	50.0	50.0	50.0
Pea	10.0	10.0	10.0
Dicalcium phosphate	19.0	19.0	19.0
Calcium carbonate	15.0	12.0	12.0
Sodium bicarbonate	2.5	2.5	2.5
Sodium chloride	2.5	2.5	2.5
DL Methionine	2.5	2.5	2.5
Lysine HCl	1.5	1.5	1.5
Vitamin mineral premix	5.0	5.0	5.0

The litters of each group have been checked weekly for faeces compactness using an arbitrary but comparative score: 0, dry litter; 1, medium wet; 2, wet. At the 36th day, the animals were sacrificed at a slaughterhouse.

**Table 4:** Nutritional traits of basal diets according to growing periods.

Item	Starter (0–12 d)	Grower (13–21 d)	Finisher (22–35 d)
Dry matter, g/kg	884	866	869
Crude protein, g/kg on DM	225	200	188
Ether extract, g/kg on DM	60	65	82
NDF, g/kg on DM	28	28	32
Ash, g/kg on DM	63	54	49
Calcium, g/kg on DM	8	7	6
Phosphorus, g/kg on DM	6	5	4
Lysine, g/kg on DM	13	12	11
Methionine, g/kg on DM	6	4	3
Metabolizable Energy, kcal/kg ^1^ on DM	2950	3010	3090

^1^ Estimated from feed tables according to Sauvant et al. (2004).

**Table 5:** Rate of microbial growth in the in vitro assay (data are reported as Log10 (CFU/mL)/h).

Pathogen	Tested Compounds ^1^						SEM ^2^	*p* ^3^
	Δ_time_^4^	C	T	S	TS	ST		
*C. perfringens*	0.5–3 h	0.186 ^a^	−0.446 ^d^	−0.096 ^b^	−0.323 ^c^	−0.032 ^b^	0.025	<0.0001
	3–24 h	0.281 ^a^	0.015 ^e^	0.131 ^b^	0.035 ^d^	0.054 ^c^	0.001	<0.0001
*S. typhymurium*	0.5–3 h	0.764 ^a^	0.039 ^e^	0.298 ^c^	0.186 ^d^	0.338 ^b^	0.011	<0.0001
	3–24 h	0.143 ^a^	0.022 ^c^	0.043 ^b^	0.029 ^d^	0.022 ^c^	0.001	<0.0001
*E. coli*	0.5–3 h	0.665 ^a^	0.192 ^d^	0.382 ^b^	0.383 ^b^	0.449 ^b^	0.028	<0.0001
	3–24 h	0.210 ^a^	0.074 ^d^	0.188 ^b^	0.080 ^d^	0.104 ^c^	0.003	<0.0001
*C. jejuni*	0.5–3 h	0.344 ^a^	−0.336 ^c^	−0.275 ^cb^	−0.254 ^cb^	−0.175 ^b^	0.048	<0.0001
	3–24 h	0.071 ^b^	0.077 ^b^	0.100 ^a^	0.086 ^ab^	0.083 ^b^	0.005	0.0219

^1^ C, control; T, 3.0 g/kg chestnut tannin; S, 3.0 g/kg of Sn1-monoglycerides; TS, 2.0 g/kg of chestnut tannin added to 1.0 g/kg of Sn1-monoglycerides; ST, 1.0 g/kg of chestnut tannin added to 2.0 g/kg of Sn1-monoglycerides. ^2^ SEM, Standard Error Mean. ^3^ Probability of significant effect due to experimental factors; ^a,b,c,d,e^ within a row, means with different Latin letters are significantly different (*p* < 0.05). ^4^ Time range considered for growth rate calculation.

**Table 6:** Live performance of birds.

Item	Diets ^1^					SEM ^2^	*p* ^3^
	C	T	S	TS	ST		
**0–14 Days**							
Weight gain, g	299.85	325.90	303.15	320.60	318.70	34.69	0.7850
Feed intake, g	526.00	571.50	502.25	509.25	538.25	68.34	0.8535
Feed/gain ratio	1.82	1.77	1.66	1.65	1.72	0.08	0.9248
**15–21 Days**							
Weight gain, g	440.50	453.75	483.50	467.25	416.25	34.69	0.7850
Feed intake, g	498.25	555.25	558.00	537.50	585.75	68.34	0.8535
Feed/gain ratio	1.32	1.27	1.20	1.20	1.24	0.08	0.9248
**22–35 Days**							
Weight gain, g	1487.50	1491.50	1497.00	1418.50	1479.75	34.69	0.7850
Feed intake, g	2179.50	2169.00	2145.00	2095.00	2,307.50	68.34	0.8535
Feed/gain ratio	1.52	1.47	1.46	1.56	1.60	0.08	0.9248
**Whole Period**							
Weight gain, g	2227.85	2271.15	2283.65	2206.35	2214.70	48.60	0.7304
Feed intake, g	3203.75	3295.75	3205.25	3141.75	3331.50	68.34	0.8535
Feed/gain ratio	1.45	1.46	1.41	1.44	1.52	0.04	0.4105

^1^ C, control; T, 3.0 g/kg chestnut tannin; S, 3.0 g/kg of Sn1-monoglycerides; TS, 2.0 g/kg of chestnut tannin added to 1.0 g/kg of Sn1-monoglycerides; ST, 1.0 g/kg of chestnut tannin added to 2.0 g/kg of Sn1-monoglycerides. ^2^ SEM, Standard Error Mean. ^3^ Probability of significant effect due to experimental factors.

**Table 7:** Major carcass traits of slaughtered birds.

Carcass Trait	Diet ^1^					SEM ^2^	*p* ^3^
	C	T	S	TS	ST		
Dressing out, %	86.27	84.68	84.65	85.35	86.27	0.79	0.4687
Live weight, g	2868	2624	2627	2560	2760	143	0.5996
Carcass weight, g	2800	2503	2504	2452	2659	151	0.5250
Eviscerate weight, g	2465	2223	2224	2185	2384	139	0.5919
Breast, g	581	527	504	516	615	67	0.7474
Tights, g	551	513	502	497	514	31	0.7655
Liver, g	78 ^a^	65 ^b^	59 ^b^	72 ^a^	82 ^a^	2	0.0023

^1^ C, control; T, 3.0 g/kg chestnut tannin; S, 3.0 g/kg of Sn1-monoglycerides; TS, 2.0 g/kg of chestnut tannin added to 1.0 g/kg of Sn1-monoglycerides; ST, 1.0 g/kg of chestnut tannin added to 2.0 g/kg of Sn1-monoglycerides. Concentration are expressed as *w*/*v*. ^2^ SEM, Standard Error Mean. ^3^ Probability of significant effect due to experimental factors; ^a,b^ within a row, means with different letters are significantly different (*p* < 0.05).

**Table 8:** Colour traits of breast meat.

Item ^2^	Diet ^1^					SEM ^3^	*p* ^4^
	C	T	S	TS	ST		
L*	53.01 ^c^	57.32 ^bc^	63.80 ^a^	58.36 ^b^	55.91 ^bc^	2.21	0.0435
A*	5.37	3.58	5.60	4.46	6.04	1.14	0.3689
B*	3.47 ^b^	3.32 ^b^	8.99 ^a^	3.56 ^b^	2.74 ^b^	1.04	0.0041

^1^ C, control; T, 3.0 g/kg chestnut tannin; S, 3.0 g/kg of Sn1-monoglycerides; TS, 2.0 g/kg of chestnut tannin added to 1.0 g/kg of Sn1-monoglycerides; ST, 1.0 g/kg of chestnut tannin added to 2.0 g/kg of Sn1-monoglycerides. Concentration are expressed as *w*/*v*. ^2^ Data are reported in the L* a* b* color notation system with L* axis representing lightness, the a* axis representing the red-green color axis (redness) and the b* axis representing the blue-yellow (yellowness) color axis. ^3^ SEM, Standard Error Mean. ^4^ Probability of significant effect due to experimental factors; ^a, b^ within a row, means with different letters are significantly different (*p* < 0.05).

**Table 9:** Calculated ΔE2000 values for breast.

	C	T	S	TS	ST
C	-	4.5565	10.5648	5.1233	2.9569
T	4.5565	-	7.2859	1.4070	3.1987
S	10.5648	7.2859	-	6.3161	8.5273
ST	5.1233	1.4070	6.3161	-	2.9534
TS	2.9569	3.1987	8.5273	2.9534	-

C, control; T, 3.0 g/kg chestnut tannin; S, 3.0 g/kg of Sn1-monoglycerides; TS, 2.0 g/kg of chestnut tannin added to 1.0 g/kg of Sn1-monoglycerides; ST, 1.0 g/kg of chestnut tannin added to 2.0 g/kg of Sn1-monoglycerides.

**Table 10:** Oxidative status of meat.

Item ^2^	Diet ^1^					SEM ^3^	*p* ^4^
	C	T	S	TS	ST		
ABTS	0.140	1.194	1.239	0.715	0.804	0.1330	0.1127
DPPH	0.188	0.228	0.224	0.158	0.183	0.0157	0.0983
FRAP	0.264	0.280	0.282	0.261	0.246	0.0141	0.4160
TBARS	0.134	0.134	0.130	0.137	0.138	0.0017	0.1469

^1^ C, control; T, 3.0 g/kg chestnut tannin; S, 3.0 g/kg of Sn1-monoglycerides; TS, 2.0 g/kg of chestnut tannin added to 1.0 g/kg of Sn1-monoglycerides; ST, 1.0 g/kg of chestnut tannin added to 2.0 g/kg of Sn1-monoglycerides. ^2^ ABTS and DPPH are expressed as mmol of Trolox equivalent per kg of meat; FRAP as mmol of Fe^++^ equivalent per kg of meat; TBARS as mg of MDA-Eq per kg of meat. ^3^ SEM, Standard Error Mean. ^4^ Probability of significant effect due to experimental factors; a, b within a row, means with different letters are significantly different (*p* < 0.05).
